# Human basal body basics

**DOI:** 10.1186/s13630-016-0030-8

**Published:** 2016-03-14

**Authors:** Anastassiia Vertii, Hui-Fang Hung, Heidi Hehnly, Stephen Doxsey

**Affiliations:** Program in Molecular Medicine, University of Massachusetts Medical School, Worcester, MA USA; Department of Cell and Developmental Biology, SUNY Upstate Medical University, Syracuse, NY USA

**Keywords:** Basal body, Cilium, Centrosome, Ciliopathy, Human

## Abstract

In human cells, the basal body (BB) core comprises a ninefold microtubule-triplet cylindrical structure. Distal and subdistal appendages are located at the distal end of BB, where they play indispensable roles in cilium formation and function. Most cells that arrest in the G_0_ stage of the cell cycle initiate BB docking at the plasma membrane followed by BB-mediated growth of a solitary primary cilium, a structure required for sensing the extracellular environment and cell signaling. In addition to the primary cilium, motile cilia are present in specialized cells, such as sperm and airway epithelium. Mutations that affect BB function result in cilia dysfunction. This can generate syndromic disorders, collectively called ciliopathies, for which there are no effective treatments. In this review, we focus on the features and functions of BBs and centrosomes in *Homo sapiens.*

## Basal body origin and basal body/centrosome cycle

Most cell types in humans have a single primary cilium that protrudes from the cell surface when the cell arrests in the G_0_ cell cycle stage. The basal body (BB) forms the base of the cilium and arises from the mother centriole of the centrosome [1, 2]. When a cell exits the cell cycle, the mother centriole docks at the plasma membrane and converts into a BB for primary cilium formation [2, 3]. Primary cilium formation is a dynamic process that can be reverted under mitogenic conditions. Cilia disassembly is a poorly understood process that occurs when the cell re-enters the cell cycle. Two pathways are involved in this process, namely Nek2–Kif24 and AuroraA–HDAC6 [4]. When the cell re-enters the cell cycle, BBs relinquish their function at the base of cilia, and convert to centrosomes/spindle poles [5, 6].

Some specific cell types grow multiple motile cilia that beat synchronously to direct fluid flow, and produce multiple BBs [7]. One example is the mucociliary epithelium in airways, otherwise known as the mucociliary escalator. The escalator covers most of the bronchi, bronchioles, and nose, and functions in continuous beating to push unwanted microorganisms and mucus up and out into the throat [8]. Little is known about the mechanism for construction of a BB in multiciliated cells. What is known is that in proliferating cells, centrioles duplicate only once per cell cycle, whereas in multiciliated cells, hundreds of centrioles form almost simultaneously in a de novo pathway. However, a recent study identified an intriguing asymmetry in this pathway: about 90 % of centrioles were synthesized from the daughter centriole of the original centrosome [9]. BBs in these cells are thought to derive from a centrosome-like opaque cytoplasmic structure called the “deuterosome.” Two molecular players implicated in this function include the protein Ccdc78 and Ccdc67, and the known centrosome proteins Cep152, Plk4, and SAS-6 [10]. From an evolutionary perspective, all metazoans rely on cytoplasmic de novo BB biogenesis for multiciliation [7]. The importance of de novo BB biogenesis in humans is illustrated in patients mutant for cyclin O. When this regulator of de novo BB biogenesis is mutated, patients exhibit progressive defects in the respiratory tract but lack the classical ciliopathy phenotype [11, 12].

During spermatogenesis, BBs are produced together with sperm metamorphosis in an interesting way. In *Homo sapiens*, round spermatids undergo a complex differentiation process that results in mature spermatozoa. In spermatozoa, the sole function of the centriole is seemingly to template the motile cilium/flagellum. Since spermatids will not enter a new mitotic cycle, their centrosomes undergo a functional shift to BBs that serve as templates for the assembly of the flagellum. Centrosome reduction then occurs. This process includes loss of the pericentriolar material (PCM) and the ability to nucleate microtubules [13, 14]. All together, humans possess a complex arsenal of mechanisms to regulate the BB, although the idiosyncrasies between cell types that regulate these processes are unknown.

## Review

### Basic basal body structure and sub-structures

**Fig. 1:**
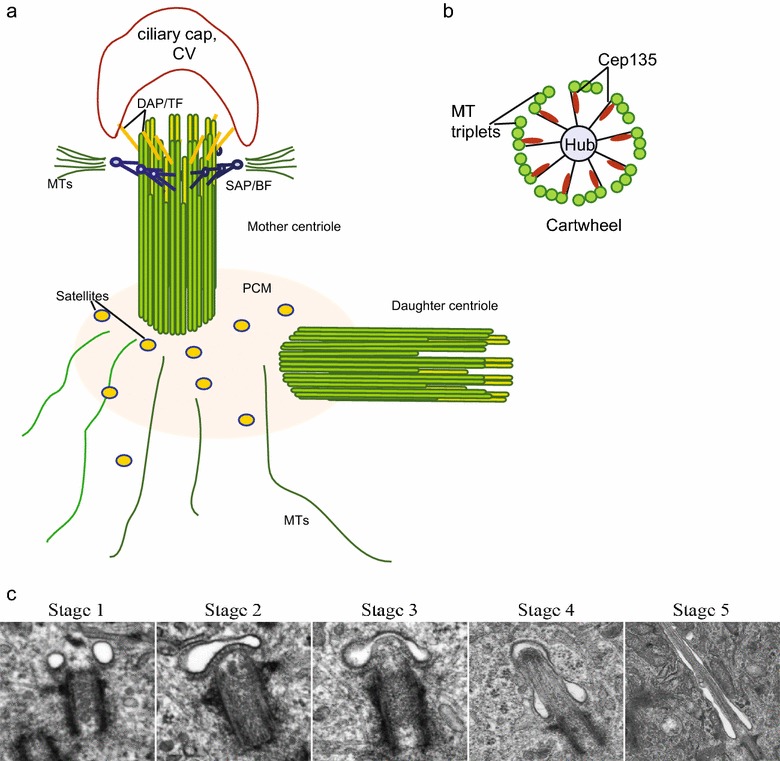
Structure of the human basal body (BB) at initial step of ciliogenesis. **a** Side view of the BB. DAP/TF, distal appendages/transition fibers, SAP/BF, sub-distal appendages/basal feet, CV, ciliary vesicle. **b** Cross section of the BB with SAS protein-containing central hub, attached to Cep135 and MT triplets. **c**. Primary ciliogenesis progresses through five morphologically distinct stages in human astrocytes. Stage 1: lateral vesicles are at the distal end of the BB. Stage 2: the lateral vesicles fuse and become a vesicular cap. Stage 3–4: stretch of vesicular cap and outgrowth of primary cilium. Stage 5: mature primary cilium surrounded with cilium pit [64]. Used with permission from [64]

The mother centriole of the centrosome serves as a physical template for human cilia formation (reviewed by Bornens 2012 [15]). The centrosome consists of a pair of MT-based centrioles (the mother/older and daughter), pericentriolar material, and pericentriolar satellites [16] (Fig. 1a). The centriole consists of 9 triplet microtubules on its proximal end, and 9 doublet microtubules on its distal end [17–20]. At the center of the centriole is a cartwheel structure with a central hub, which organizes the ninefold symmetric MT centriole wall. CEP135 at the centriole wall links with SAS-6 at the cartwheel hub [21] (Fig. 1b). Distal ends of the BBs/mother centrioles posses two sets of appendages, namely distal (DAP) and sub-distal (SAP) appendages. Human BBs and centrosomes contain five types of tubulin: *α, β, γ, δ*, and *ε* [22]. While MT polymers consist exclusively of α- and β-tubulin heterodimers; γ-tubulin is integrated into *γ*-tubulin ring complexes (γ-TURCs), which are responsible for MT nucleation [23–26]. *ε*-Tubulin associates with sub-distal appendages of the centrioles and is critical for centriole duplication and MT organization [27, 28].

DAPs (also called “transition fibers” in cilia) dock BBs at the plasma membrane and initiate ciliogenesis [29–31]. DAPs initiate ciliogenesis by mediating the formation of the ciliary vesicle through Rab GTPases [32] and IFT20 [33], both of which are important vesicle trafficking components [34–36]. C2cd3, which localizes to the distal end of the BB, is required for DAP formation [37]. During DAP assembly, Cep83 is required for recruitment of multiple DAP proteins including Cep89 (Cep123), SCLT2, FBF1, and Cep164 [30]. Cep164 is a multifunctional DAP protein that orchestrates several events during early ciliogenesis. For example, Cep164 is indispensible for ciliary vesicle formation [38, 39], and BB docking at the plasma membrane [29, 38]. Moreover, Cep164 directly recruits tau tubulin kinase-2 (TTBK2) to the BB [40], where it is critical for CP110 removal from the BB—an important prerequisite for ciliogenesis [41, 42]. These observations suggest that Cep164 mediates not only the BB-membrane docking step, but also coordinates ciliogenesis. In addition to Cep164, Cep89 (Cep123) participates in ciliary vesicle formation [43]. Consistent with a DAP role in ciliogenesis is the evidence that mutations in DAP proteins such as C2cd3 [44], Cep83 [45], Cep164 [46], and SCLT1 [47] result in ciliopathies.

SAPs (also called “basal feet” in cilia) are involved in MT anchoring [48] (Fig. 1a), and were not considered to be involved in cilia function until recently. (1) Mutations in SAP proteins have now been shown to cause ciliopathies [49–51]. (2) The SAP proteins, cenexin and centriolin, are specifically required for recycling endosome trafficking and ciliogenesis [34, 52, 53]. (3) SAPs and the ciliopathy protein complex, the BBSome [54] are connected in the sense that BBS4 is required for MT anchoring. The BBSome is a 7-protein complex that is associated with the ciliopathy, Bardet-Biedl syndrome [55]. Based on this evidence, SAPs, as BB sub-structures, may be involved in ciliary functions.

Pericentriolar satellites are dynamic dynein and kinesin-driven electron-dense granules located within and around the pericentriolar material (PCM) [56, 57]. Satellites consist of dozens of proteins, many of which are required for cilia formation [57]. This suggests that satellites modulate ciliogenesis, although their precise role in this process remains elusive [36, 57]. Recent evidence suggests that the satellite proteins, Cep290 and PCM1, are involved in ciliogenesis through modulating Rab8 recruitment to BBs [58, 59]. In addition, recent studies demonstrate that autophagy, a process that turns over cellular debris, can promote ciliogenesis by degrading select centriolar satellite proteins such as OFD1 [60, 61]. OFD1 is localized to SAPs and pericentriolar satellites and is responsible for recruitment of Cep290 to these sites [62]. These results suggest that satellites are active BB substructures that contribute to ciliopathy pathogenesis when disrupted [63].

Another transient BB substructure, the ciliary vesicle (Fig. 1a, c), appears first as small vesicles that accumulate at DAPs of the BB before primary cilia formation. These vesicles appear to fuse to form a ciliary vesicle “cap.” The BB and associated cap is thought to move up to and fuse with the plasma membrane allowing the cilium to grow and extend out into the extracellular space. A ciliary pit is created after the cilium is fully made through an interaction between the ciliary vesicle membrane and DAPs [64] (Fig. 1c, stages 4 and 5). Upon exit from G_0_ and primary cilia disassembly, cilia components and cilia membrane are inherited by the mother centriole. Strikingly, these components seem to be retained at the oldest spindle pole (the pole containing the mother/oldest centriole) when the cell divides again [65]. The daughter cell that contains the oldest spindle pole and the inherited ciliary membrane components re-establishes a primary cilium earlier than the cell that lacks these ciliary components. These studies imply that the centrosome-associated ciliary membrane functions in temporal control of ciliogenesis [65].

During cilia assembly, the BB facilitates formation of the ciliary rootlet [66]. This structure is formed by oligomers of the protein, rootletin, [67–69], which provides support for the cilium. Besides its function at the base (proximal end) of the BB, rootletin is also a component of the centrosome during G1 and S cell cycle stages and is required for centrosome cohesion [70]. Taken together, human BBs are equipped with transition fibers (DAPs), the ciliary rootlet, and basal feet (SAPs) [22].

## Identification of basal body components

Proteomic analyses of human centrosomes have uncovered many centrosome-associated proteins [71, 72]. BB components were identified in these studies as well as in the cilia proteome [73], in expression studies from cilia in ciliopathy patients [74], and in the spermatozoan proteome [75]. A number of mother-centriole-specific proteins were identified using PCP-SILAC mass spectrometry. Ccdc41 and Cep89 are two recent examples [71]. A latter study confirmed their DAP localization, and their critical roles in ciliary docking to the plasma membrane and subsequent cilia formation [30]. Moreover, a cilia proteomic database, Cildb, is a useful resource for comparing BBs, centrioles, and centrosomes across different organisms [76, 77].

## Other functions of the basal body

BBs possess most of the characteristics of centrosomes, including the ability to organize the microtubule cytoskeleton. It appears that one of the major regulatory roles of BBs is coordination of several complicated trafficking pathways. One example is a sub-compartment of the endocytic pathway, called the recycling endosome. Two GTPases are involved in its organization and function, namely Rab8 and Rab11, which are also reported to have an association with the centrosome [34, 53]. Strikingly, these same GTPases have been implicated in early stages of ciliogenesis through a Rab-GTPase cascade [78]. Recycling endosome vesicles modulated by Rab11 are brought to the basal body with the Rab8 guanine nucleotide exchange factor (GEF), Rabin8. It is proposed that once Rab11 vesicles with Rabin8 accumulate at the centrosome, Rabin8 activates Rab8 to initiate ciliogenesis. Two additional known regulators of endocytosis, EHD1 and EHD3, associate with this cascade and influence ciliary vesicle formation at DAPs [32]. These studies suggest that during cilia formation, the centrosome usurps a handful of regulatory proteins to manufacture a cilium.

In addition to GTPase modulation of cilia, proteasome-mediated protein degradation is another mechanism by which BBs/centrosomes influence ciliogenesis. Although proteasomes are distributed throughout the cell, specific biological functions of the proteasome directly at centrosomes have been reported [79]. In mammalian neurons, proteasomes localized at the centrosome regulate degradation of local ubiquitin conjugates promoting the elaboration of dendrite arbors [80]. Centrosome-localized proteasomes are also responsible for centrosome deconstruction during fever [81]. Recently, BB-localized proteasomes were implicated in ciliogenesis by removal of a negative regulator of ciliogenesis, trichoplein [82]. However, the mechanism of proteasome recruitment to the centrosome and/or BB is unknown.

Although BBs are best defined by their role as the template for cilia formation, they also function in non-ciliated human cells. For example, in lymphocytes, the centrosome docks to the plasma membrane via DAPs to form an immune synapse in much the same way as BBs dock to the plasma membrane to form cilia. Depletion of CP110, a negative regulator of ciliogenesis, and its concomitant removal from the mother centriole induces ciliogenesis in these cells, providing evidence that the centrosome at this step is transformed into a BB [83]. In this capacity, BBs facilitate IFT-dependent transport of T-cell receptors to the synapse, and mediate cytolytic granule release into the target cell [84–86].

## Notable basal body findings

Sorokin was among the first to demonstrate the need for basal bodies to interact with membranes and for microtubule growth to be coordinated with membrane extension during ciliogenesis [87]. This interaction between BBs with the plasma membrane requires the Rab GTPase cascade and membrane-shaping proteins [32]. Another significant step in basal body biology was the identification of a great number of human disorders, namely ciliopathies and brain-related disorders, like microcephaly, that are associated with mutations in BB components [11, 51, 74, 88–98]. This, in turn, was paralleled by the realization that centrosome proteins are essential for cilia formation and integrity [29, 31, 99]. Taken together, these findings lay the groundwork for understanding the molecular mechanisms of BB function that contribute to ciliopathies.

## Conclusions

### Strength and future of basal body research in humans

Essential efforts toward identification of additional mutations in centrosome/BBs that cause ciliopathies exponentially expand our current knowledge on centrosomes/BBs. This will both facilitate our understanding of these important structures and, in turn, will help in the design of new therapies for ciliopathies, which currently cannot be cured. For example, obesity and impaired ciliogenesis are key features for patients with BBS. During adipocyte differentiation, a transient primary cilium is formed, and the Wnt and Hedgehog receptors present on this primary cilium can inhibit adipogenesis. This has important implications for patients with BBS, where obesity is perhaps caused through impaired ciliogenesis and Wnt/Hedgehog signaling. Moreover, the activity of adipogenic glycogen synthase kinase 3 (GSK3) is enhanced in BBS patients because Wnt signaling is not available to antagonize it. Therefore, pharmacological inhibition of GSK*β* could become a potential treatment for BBS patients [100].

However, the overlapping properties and functions of centrosomes and BBs, and the ability of both to perturb ciliogenesis when disrupted, make it difficult to discern the molecular mechanisms behind ciliopathies. Moreover, it remains to be determined if cilia, centrosomes, and BBs all contribute to the etiology of these disorders [36] and if so, to what extent. Finally, other functions of BBs and centrosomes must be considered in the context of these disorders such as mitotic defects that are caused by cilia proteins [101].

## Abbreviations

BB: basal body
BBS: Bardet-Biedl Syndrome
DAP: distal appendages
IFT: intraflagellar transport
MTs: microtubules
MTOC: microtubule organizing center
PCM: pericentriolar material
SAP: sub-distal appendages
TCR: T cell receptor
